# The importance of the olfactory sense in the human behavior and evolution

**Published:** 2009-04-25

**Authors:** C Sarafoleanu, C Mella, M Georgescu, C Perederco

**Affiliations:** ‘Sfanta Maria’ Clinical Hospital, ENT – HNS Department, BucharestRomania

## Abstract

Not long ago it was believed that the human olfactory sense had a low importance, a vision which turned into the exploration of the environment. Recent studies have shown that, despite the weak representation of the olfactory receptor common in other species too, the cortical areas of integration of the olfactory sensations are very large and have important interconnections with memory, language, and neuro–vegetative areas.

In humans, olfaction has a small contribution in identifying objects or other people, but plays an important social and emotional part. People learn to love or to hate certain foods or objects only by appreciating their odor and this proved to be a very important economic factor.

The most significant role of olfactory signals in humans appears to be the modulation of their behavior and interpersonal relationships, of their affiliation to certain groups or social classes, having a major influence in their tastes and personality.

## Introduction

Olfactory sense is, in terms of evolution, one of the oldest senses, allowing the organisms with receptors for the odorant to identify food, potential mating partners, dangers and enemies. For most living creatures and for mankind smell is one of the most important ways of interaction with the environment.

The olfactory area in humans is about 2.5 cm^2^ wide and contains a number of about 50 million receptor cells with 8–20 cilia down in a layer of mucus of about 60 microns thick, produced by Bowmann glands in the olfactory epithelium. [1]. Only volatile substances, soluble in mucus, can reach the receptors and interact with them and finally produce sensations. The olfactory epithelium contains a variable number of basal cells, which are capable of mitotic division giving rise to mature receptor neurons. The olfactory neuron's turn over is about 40 to 60 days [1]. The axonal ramifications of these neurons go together in groups of 10 to 100 fibers, they cross the ethmoid cribriform plate reaching the olfactory bulb where they converge and form synaptic structures called glomeruli and then they converge again to mitral cells. The total convergence ratio is 1000:1 and has the important role of increasing the sensibility of the olfactory signal that will be sent to the specialized areas in the brain [2].

The olfactory epithelium contains another sensitive system via sensitive branches of trigeminal nerve. Many odorants cam produce sensations transmitted by trigeminal nerve. For example levo–menthole. If placed in the nasal cavity it produces cold sensations in small amounts and hot sensations in much bigger quantities [3]. Just the same way camphor produces a cold sensation via trigeminal nerve. Olhoff postulates that about 70% of odors also stimulate the trigeminal nerve, but with an intensity several times smaller than that of the olfactory receptors [4].

## Human olfactory sense, adaptation or involution

Humans have only 350 functional genes for olfactory receptors, compared with other mammals, e.g. mice, which have about 1.100 active genes [5]. The genes that encode the receptors are grouped in series of introns in the coding region. In mammals, these regions are organized in clusters of 10 or are often located on different chromosomes. In the human genome, there is a large amount of pseudo genes, which suggest that olfaction became less important during evolution. Recent studies showed that, in humans, more than 70% of the olfactory receptors encoding genes are actually pseudo genes, differentiating us from rats or primates which have less than 5% pseudo genes [6].

Other studies are demonstrating that humans have a good sense of smell in spite of all genetic aspects that may tend to deny this theory. Oenologists or perfume creators are capable of distinguishing thousands of odors. Human olfaction can overdue tests like gaseous chromatography in detecting the odorant molecules. All these things are realized with a small number of receptors but with the aid of certain accessory functions gained during evolution.

In the process of achieving the bipedal position, the nose and olfactory receptors have risen above the ground level and the olfactory area became smaller to allow the orbits to come closer and provide stereoscopic view. Being much further from the ground the odors received, stopped being contaminated and mixed with each other. All this, together with the air purification function of the nose, made the smells more easily to perceive and this means that the olfactory area grew smaller without many sacrifices for the olfactory sensations [1].

Once humans gained bipedal position, they began to cover large distances and to diversify their food. Then, the discovery of fire about 2 million years ago, has diversified even more the odors and taste of food. This way, odorant molecules from food reached the olfactory area by retro nasal way while eating, being processed and integrated in specialized areas in the neocortex together with the taste sensations generating flavors. In time, during evolution, beginning with livestock, plant cultivation, and spice use, more and more information were received via retro nasal way creating many complex flavors. No other species of mammals or primates could ever benefit from this kind of olfactory stimulation during their period of evolution.

During the evolution of human race, the brain increased in size and volume. The classic approach considers that olfactory organs diminished in size and function leaving vision and hearing to be the most important senses, but the theory considers only the size of the receptor area. Integration areas of olfactory sensations are extensive, including the olfactory cortex and the olfactory tubercle, some parts of cerebral tonsils, certain hypothalamic areas, medio–dorsal thalamus and medial and lateral orbito–frontal cortex [7]. The memory of discrimination and comparison between odors comes to contribute by activating areas from frontal and temporal lobes linked to the association areas introducing a new and very important cognitive component of olfaction, a component never found in other species.

Reduced repertoire of genes for olfactory receptors is compensated by the great capacity of human brain processing.

Language and speech also play an important role in the perception and discrimination of the odors. Although, we find it hard to describe an olfactory sensation, this is very important in defining and bringing the smell in the cognitive part of consciousness. The classic example of wine tasters is relevant for this process. The oenologist analyzes orthonasal and retronasal perception, compares them with other flavors from his memory, and is capable to identify constituents separately. This process is a cognitive effort including smell, language, taste, and memory [7].

Certain chemicals can induce complex unconscious neuro–vegetative reactions when stimulating the nasal receptors. This capacity was preserved during evolution from the inferior life forms until the mammals, including primates [8]. The most important are the pheromones, chemicals that play a significant role in communication, attraction, and reproduction. These substances are received and differentiated by the vomeronasal organ, which is extremely well developed in insects, snakes, or rodents. The vomeronasal organ is also present in human fetus but it has never been proven that it persisted until the adult age.

*Savic et al.* proved that women who smell some androgen–like substances have a higher activity in the preoptic and ventromedial hypothalamic nuclei. In men, estrogenic substances determine a higher activity in the paraventricular and dorsomedial hypothalamic nuclei. The different reactions could possibly have an important sexual component and reveal the possibility of vomeronasal organ persistence in the adult life [9].

*Mombaerts, Greer et al.* have proved the existence of at least one gene in the human genome that, in mice, is responsible for receptors, which are distinguishing odorless chemicals such as pheromones [10]. Although, not so long ago, these receptors were thought to be completely absent in humans, nowadays it is widely accepted that they exist in some form which has not yet been discovered.

Olfaction in humans has a role in adding emotional attributes to certain events and objects and it is not vital in the process of finding different objects from the environment and distinguishing between them. Humans use their sense of sight more, leaving smell for other purposes than exploring the environment. As an example, the smell of a banana can stimulate our appetite and it is important for this thing in particular, but it is not vital if we try to distinguish between the same banana and other objects or food. Sight is sufficient for differentiating between different objects but smell brings quality, consistence, and emotion to visual sensations. [11]

The human being is flexible, being able to learn to like certain things because of how they smell. Especially when we talk about food, flavors have a great importance proved by the existence of so many different, usual or extremely exotic, culinary combinations.

Smells and flavors are important socio-economic factors. Olfactory signals can act as a catalyst in the social interactions or can be used as marketing strategies.

Some say that ‘the nose smells what the eyes see’ because sometimes a simple chemical ingredient added can make a type of food to resemble another.The hippocampus mediates a reactivation of semantic intermodal association even in the absence of explicit memory [11].

## Conclusions

The olfactory sense could have unbelievable attributes if we consider its' capacity to modulate human behaviors. It has determinant roles in the evolution of human habitat, in the way of preparing food and, most important of all, in the social behavior.

The odor is thought to be essential in defining human inner ego as an indispensable attribute of sophistication and complexity. The odor can even allow tracing the limits between professions, races, or diseases.

Back in the ancient times, perfumes used to play a part in defining sexuality consciously contributing to the unconscious effect of pheromone–like chemicals on the vomeronasal organ.

The smell is linked to taste and appetite in the same way emotions are associated with arts and normal social life requires intense participation of all the five senses in variable proportions, depending on the situation [12].
